# Sex-Specific Association of Serum Uric Acid Level and Change in Hyperuricemia Status with Risk of Type 2 Diabetes Mellitus: A Large Cohort Study in China

**DOI:** 10.1155/2020/9637365

**Published:** 2020-07-23

**Authors:** Yanmei Lou, Pei Qin, Changyi Wang, Jianping Ma, Xiaolin Peng, Shan Xu, Hongen Chen, Dan Zhao, Li Wang, Dechen Liu, Yang Li, Ping Zhao, Dezhu Han, Dongsheng Hu, Fulan Hu

**Affiliations:** ^1^Department of Health Management, Beijing Xiaotangshan Hospital, Beijing, China; ^2^Department of Epidemiology and Health Statistics, School of Public Health, Shenzhen University Health Science Center, Shenzhen, Guangdong, China; ^3^Department of Non-Communicable Disease Prevention and Control, Shenzhen Nanshan Center for Chronic Disease, Shenzhen, Guangdong, China; ^4^Department of Epidemiology and Health Statistics, School of Public Health, Zhengzhou University, Zhengzhou, Henan, China; ^5^Beijing Fangshan District Yanshan Commission of Culture, Health and Family Planning, Beijing, China

## Abstract

**Background:**

Conflicting findings have been reported regarding the sex-specific association between serum uric acid (SUA) level and type 2 diabetes mellitus (T2DM) risk, and no study has explored the association between the change in hyperuricemia status and T2DM risk. The study was aimed at exploring the sex-specific association of baseline SUA and changes in hyperuricemia status with T2DM risk.

**Methods:**

We included 37,296 eligible adults without T2DM at the first examination who attended the baseline examination and at least one follow-up annual examination. Cox and logistic regression models were used to calculate hazard ratios (HRs) and odds ratios (ORs) with their 95% confidence intervals (CIs) for T2DM risk associated with baseline SUA and the change in hyperuricemia status, respectively.

**Results:**

During a median follow-up of 3.09 years, of 37,296 eligible adults, 2,263 developed T2DM. Compared with the first SUA quartile, higher quartiles were associated with an increased risk of T2DM in women (HR 1.78, 95% CI 1.17-2.71 for Q3 and 1.93, 1.27-2.93 for Q4; *P*_trend_ < 0.001) but not in men. Compared with women with a persistent normal SUA level at baseline and the last follow-up, T2DM risk increased significantly among those whose SUA status changed from normal at baseline to hyperuricemia at the last follow-up (OR 1.71, 95% CI 1.12-2.55) and those with persistent hyperuricemia at baseline and the last follow-up (OR 2.37, 95% CI 1.60-3.46). However, for men, a nonsignificant association was found between the change in hyperuricemia status and T2DM risk.

**Conclusions:**

Baseline SUA and the change in hyperuricemia status were associated with T2DM risk only among women. The findings suggest the importance of monitoring SUA levels and maintaining them within a normal range for preventing or reducing incident T2DM in Chinese women.

## 1. Introduction

Type 2 diabetes mellitus (T2DM) is considered a public health concern worldwide. The International Diabetes Federation estimated that the number of T2DM patients worldwide was 425 million in 2017 and was expected to increase to 629 million by 2045 [1]. China has the largest number of people with T2DM, with an estimate of 114 million in 2017 [1]. Therefore, identifying and clarifying the risk factors for T2DM are crucial for the implementation of primary prevention.

Serum uric acid (SUA), an end product of the metabolism of purine nucleotides, has been suggested as a risk factor for T2DM in an increasing number of epidemiological studies [2–6]. To our knowledge, some cohort studies have investigated the association but with relatively small sample sizes ranging from 711 to 12,643 and inconsistent findings reported [2–5, 7–14]. Evidence on the sex-specific differences in the SUA level-T2DM risk association is limited and has resulted in conflicting findings [2, 5, 6, 12, 15, 16], with studies showing a significant association in both sexes [12], only women [2], or only men [4, 5, 15]. Therefore, more studies are warranted to clarify the sex-specific association between SUA level and T2DM risk.

Animal experiments [17] and few intervention studies in humans [18] have shown that reducing uric acid might improve insulin resistance. This raises great interest in whether dynamic changes in SUA levels under nonintervention conditions can affect T2DM risk. However, research is lacking on whether changes in hyperuricemia status have an effect on the risk of T2DM.

Therefore, we investigated the sex-specific differences in the association between SUA level and T2DM risk and further explored whether the change in hyperuricemia status was associated with T2DM risk in a large Chinese longitudinal cohort.

## 2. Materials and Methods

### 2.1. Study Population

This cohort analysis included adults aged ≥18 years who underwent a comprehensive annual health examination at the clinics of Xiaotangshan Hospital, Beijing, from 2009 to 2016. Adults without T2DM at entry, who attended at least one follow-up visit and had complete data on any indicators for the diagnosis of T2DM at entry and follow-up, were evaluated for eligibility (*n* = 41,439). If participants attended more than one follow-up, we only chose the data of the last follow-up for analysis, thus leaving the data that were collected at baseline and the last follow-up in the present study. Among participants, those with a history of myocardial infarction, stroke, coronary heart disease or heart failure (*n* = 1,393), cancer (*n* = 456), and estimated glomerular filtration rate (eGFR) < 60 mL/min/1.73 m^2^ at baseline (*n* = 1,446) were excluded. After excluding adults with missing data for SUA at baseline (*n* = 121) and at the last follow-up (*n* = 727), a total of 37,296 adults were enrolled in the study, with a median follow-up of 3.09 years. The study was approved by the Institutional Review Board of Xiaotangshan Hospital (no. 202006).

### 2.2. T2DM Diagnosis and SUA Criteria

According to the criteria of the American Diabetes Association [19], T2DM was defined as a fasting glucose level ≥ 126 mg/dL, self-report of a physician's diagnosis of diabetes, or the use of antidiabetes treatment. Baseline SUA levels were divided into sex-specific quartiles (quartiles 1-4): ≤5.44, >5.44~≤6.22, >6.22~≤7.08, and >7.08 mg/dL for men and ≤3.80, >3.77~≤4.38, >4.38~≤5.05, and >5.05 mg/dL for women. Hyperuricemia was defined as an SUA level ≥ 7.0 mg/dL for men and ≥6.0 mg/dL for women [20]. SUA was measured at baseline and at every annual check-up. Among 38,023 eligible participants with a baseline measurement of SUA, 37,296 had repeated measurements at the last follow-up. According to the presence of hyperuricemia at baseline and the last follow-up, participants were categorized into four groups separately for men and women: persistent normal SUA level at both baseline and the last follow-up, hyperuricemia at baseline and normal SUA level at the last follow-up, normal SUA level and hyperuricemia at the last follow-up, and persistent hyperuricemia at both baseline and the last follow-up.

### 2.3. Covariates

Information on the demographic characteristics (age and sex), medical history, and use of medications was collected through in-person standardized questionnaire interviews. All participants also underwent a comprehensive clinical and biochemical examination. Height, weight, and waist circumference (WC) were measured by well-trained nurses using standard methods, with participants wearing light clothes and no shoes. The body mass index (BMI) was calculated as weight in kilograms divided by height squared in meters. According to the Working Group on Obesity in China (WGOC), general overweight/obesity was defined as BMI ≥ 24 kg/m^2^ [21]. Abdominal obesity was defined as WC ≥ 90 cm for men and WC ≥ 80 cm for women according to the International Diabetes Federation Epidemiology Task Force Consensus Group [22]. Systolic and diastolic blood pressure was measured twice by trained nurses using an electronic sphygmomanometer (HEM-770AFuzzy, Omron, Japan) on the right arm at the heart level in participants who were in a seated position after at least 5 min of rest. Hypertension was defined as systolic blood pressure ≥ 140 mmHg or diastolic blood pressure ≥ 90 mmHg or current use of antihypertension medication according to the National High Blood Pressure Education Program [23]. Overnight fasting blood samples were obtained. Serum concentrations of uric acid, total cholesterol (TC), triglycerides (TG), high-density lipoprotein cholesterol (HDL-C), low-density lipoprotein cholesterol (LDL-C), and creatinine were measured by an enzymatic colorimetric assay (Type 7600; Hitachi, Tokyo). Fasting plasma glucose (FPG) and alanine aminotransferase (ALT) levels were measured by using an automated analyzer. The value of eGFR was calculated with the following equation: eGFR (mL/min/1.73 m^2^) = 175 × creatinine^−1.234^ × age^−0.179^ [if women, ×0.79], where creatinine is in mg/dL and age is in years [24].

### 2.4. Statistical Analyses

Baseline characteristics of the study population are described as quartiles (Q1-Q4) of SUA. Continuous variables are presented as the mean (SD) or median (interquartile range) if nonnormally distributed; categorical variables are presented as frequency (%). ANOVA, Kruskal-Wallis *H* test, and chi-square test were used to analyze the differences across quartiles of SUA for continuous data, skewed continuous data, and categorical data, respectively.

The study endpoint was the diagnosis of T2DM. Cox proportional hazards models were used to calculate hazard ratios (HRs) and 95% confidence intervals (CIs) for the association between baseline SUA quartiles and T2DM, comparing sex-specific quartiles of baseline SUA with the first quartile as a reference. An increasing number of covariates were adjusted as follows: model 1 was adjusted for age at baseline; model 2 was further adjusted for BMI, WC, heart rate, systolic blood pressure, and levels of TC, TG, HDL-C, eGFR, ALT, and white blood cell count at baseline; and model 3 was further adjusted for FPG at baseline. The proportional hazards assumption was tested by using scaled Schoenfeld residuals [25]. If variables violated the proportional hazards assumption, a time-dependent covariate was constructed using the time-transform functionality of *coxph* by R programming in the Cox proportional hazards model [26]. *P*_trend_ across quartiles of SUA was evaluated by imputing the median value within each SUA quartile as a continuous variable in Cox proportional hazards models. Subgroup analyses were conducted by stratification according to baseline characteristics, including age (<50 or ≥50 years), BMI (<24 or ≥24 kg/m^2^), and hypertension status (yes or no) in the multiple Cox models. The interaction effect (*P*_interaction_) was calculated by including an interaction term in the multiple Cox models.

Logistic regression models were used to investigate the association between changes in SUA level between baseline and the last follow-up and T2DM risk, with persistent normal SUA level at both baseline and the last follow-up used as a reference. An increasing number of covariates were adjusted as follows: model 1 was adjusted for age at baseline and follow-up; model 2 was further adjusted for BMI, WC, heart rate, systolic blood pressure, and levels of TC, TG, HDL-C, eGFR, ALT, and white blood cell count at baseline; and model 3 was further adjusted for FPG at baseline. All statistical analyses were performed with R 3.5.2 (R Foundation), with two-sided *P* < 0.05 considered statistically significant.

## 3. Results

### 3.1. Demographic Characteristics of the Study Participants

During 130,509 person-years (median 3.09 years) of follow-up, 1,437 men and 505 women developed T2DM; the incidence was 18.51/1,000 person-years for men and 9.55/1,000 for women. The median age of the study population at baseline was 41 years (range 18-96), and 43.1% were women. The mean SUA level was 6.31 mg/dL (SD 1.26) for men and 4.47 mg/dL (SD 0.98) for women. Baseline characteristics of participants by SUA quartiles are shown in Table 1. Participants who had higher SUA levels were more likely to be older and had higher BMI, WC, systolic and diastolic blood pressure, FPG, TC, TG, LDL-C, creatinine, and ALT and lower levels of HDL-C than those with lower SUA levels for both men and women.

### 3.2. Sex-Specific Association between SUA Level and T2DM Risk

We estimated the association between SUA level and T2DM risk for men and women separately (Table 2). Among women, higher SUA levels were significantly associated with an increased risk of T2DM in model 3 (HR 1.78, 95% CI 1.17-2.71 for the third vs. the first quartile and 1.93, 95% CI 1.27-2.93 for the highest vs. the first quartile). The risk of incident T2DM for women significantly increased with increasing SUA levels (*P*_trend_ < 0.001). We also observed a significant association between hyperuricemia and T2DM risk among women (HR 1.35, 95% CI 1.01-1.80) as well as a significant dose-response association in which T2DM risk increased by 20% per 1 mg/dL increase in SUA level (HR 1.20, 95% CI 1.08-1.34). However, we observed no significant association between SUA and T2DM risk among men in model 3 when comparing SUA quartiles to the reference or when comparing hyperuricemia to normouricemia, and no significant dose-response association was found between SUA level and T2DM risk.

**Figure 1:**
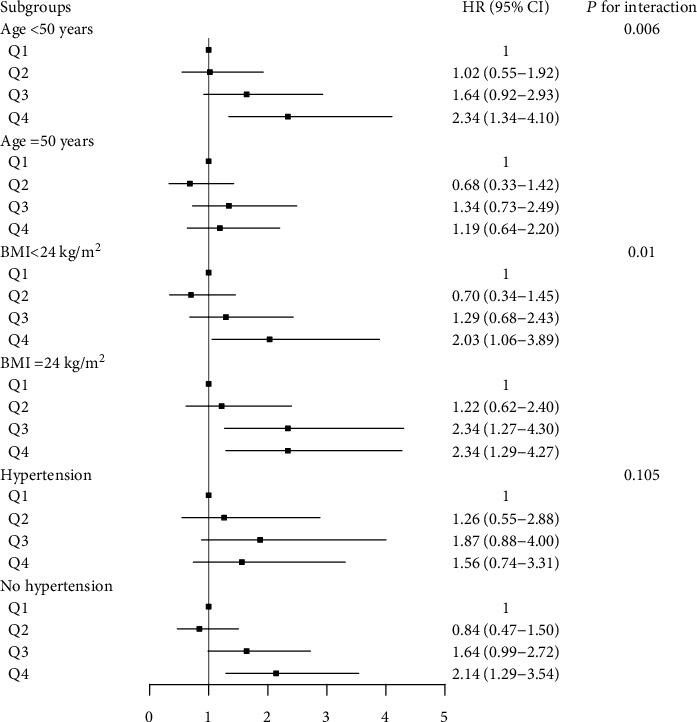


We conducted subgroup analyses for men and women separately (Figure 1). Higher SUA quartiles, especially the fourth quartile, were associated with significantly increased T2DM risk among women aged <50 years (*P*_interaction_ = 0.006), without hypertension (*P*_interaction_ = 0.010) and with BMI ≥ 24 and <24 kg/m^2^ (*P*_interaction_ = 0.105). However, we observed no significant association between SUA level and T2DM risk in the subgroup analyses for men.

### 3.3. Sex-Specific Association between Change in Hyperuricemia Status and T2DM Risk (Table 3)

For women, compared with participants who had normal SUA levels at both baseline and the last follow-up, those who had normal SUA levels at baseline and hyperuricemia at the last follow-up and those who had persistent hyperuricemia at the last follow-up had a higher risk of T2DM; the corresponding ORs for T2DM were 1.71 (95% CI 1.12-2.55) and 2.37 (95% CI 1.60-3.46), respectively, in model 3. However, no significant association was observed among men for SUA change groups in model 3.

## 4. Discussion

We found that SUA was positively associated with the risk of T2DM in the Chinese longitudinal cohort study only among women, and the association might be modified by age and BMI at baseline. We further found that T2DM risk increased significantly among women with the change from normal SUA level at baseline to hyperuricemia at the last follow-up and with persistent hyperuricemia at baseline and the last follow-up, compared with the risk associated with persistent normal SUA level at baseline and the last follow-up.

Previous studies and meta-analyses have reported the SUA level as an independent predictor of new-onset T2DM [27, 28]. The SUA level was reported to be higher among men than among women [29]; therefore, we should estimate the association between the SUA level and T2DM risk in men and women separately. We observed a significant association between SUA level and T2DM risk only among women, which was in agreement with some recent studies [2, 6]. One recent 5-year retrospective cohort study of a Korean population (*n* = 10,505) found a significant association between hyperuricemia and the risk of T2DM among women but not among men [6]. Another retrospective longitudinal study of the Japanese population (*n* = 10,717) demonstrated a significant association between SUA quartiles and the risk of T2DM only among women [2]. In contrast, three cohort studies conducted in Australia (*n* = 4,259) [15], America (*n* = 5,012) [4], and the Netherlands (*n* = 8,367) [5] found a significant association between SUA level and T2DM among men but not among women. Additionally, one prospective cohort study of a middle-aged and older Chinese population identified a significant association between SUA level and risk of T2DM for both sexes, but the sample size was relatively small (*n* = 924) [12]. The discrepancy in the sex-specific association between SUA level and T2DM risk might be explained by differences in study design, sample size, and the contribution of SUA level to the development of T2DM in Asian populations compared to that in other populations. Based on published observational studies and our results, SUA may be a risk factor for incident T2DM only for women in Asian populations.

Overweight/obesity and hypertension often cooccur with T2DM [30, 31], and they are also related to SUA levels [32]. We performed sex-specific subgroup analyses stratified by BMI and hypertension status. A significant interaction effect was found between SUA level and BMI at baseline on T2DM risk, and a higher risk was observed in women with overweight/obesity than in those without, which may be because overweight/obesity can increase the risk of T2DM and thereby aggravate the effect of SUA level on T2DM risk. A nonsignificant interaction effect was found between hypertension status and SUA level on T2DM risk, which suggested that high SUA levels increased the risk of T2DM for women, regardless of the presence of hypertension at baseline. Additionally, increased SUA levels may result from hypertension, which may mask the actual effect of SUA levels on T2DM. Moreover, we found that the significant association for women aged <50 years and the nonsignificant results for women aged ≥50 years might be explained by the reduced statistical power due to the small sample size of individuals with incident T2DM in the quartile 1 group (*n* = 20 in women aged ≥50 years).

The present study is the first to estimate the risk of T2DM associated with changes in hyperuricemia status by comparing individuals with different statuses of hyperuricemia between baseline and the last follow-up with individuals with persistent normal SUA levels at baseline and the last follow-up in a Chinese population. For women, a change from a normal SUA level to hyperuricemia and maintaining persistent hyperuricemia can significantly increase the risk of T2DM, but a change from hyperuricemia to a normal SUA level may not prevent or reduce the risk of incident T2DM. The findings suggest that monitoring SUA regularly and maintaining the level within a normal range are helpful to prevent T2DM. However, changes in SUA levels may occur because of changes in other unmeasured behavioral risk factors (e.g., physical activity, smoking, and dietary factors). More studies are needed to confirm the stability of the results. Reasons for the nonsignificant association between a change from hyperuricemia to normal SUA level and risk of incident T2DM may be the reduced statistical power due to the small sample size and the relatively short follow-up duration.

The following underlying biological mechanisms may support the plausibility of the association between SUA and T2DM. Increased SUA levels can induce oxidative stress [17, 33, 34], which has been established as a pathological pathway for the development of T2DM [35]. Oxidative stress involves the activation of nicotinamide adenine dinucleotide phosphate (NADPH) oxidase and the generation of oxidized lipids and inflammatory mediators [17, 34]. Moreover, SUA may have a direct effect on plasma glucose by inhibiting beta cell function and increasing hepatic glucose production [33, 36]. An in vitro study revealed that isolated pancreatic islets under the condition of high uric acid levels could decrease basal and glucose-induced insulin secretion [33]. High SUA levels could significantly reduce adenosine monophosphate-activated protein kinase activity, thereby increasing hepatic glucose production [36]. Epidemiological studies revealed a significant association between SUA level and B cell dysfunction only in women [37], which may explain the sex-specific difference in the association between SUA level and T2DM risk. Reproductive factors such as menopausal status, earlier age at menarche, and ever use of oral contraceptives can increase SUA levels, so the different estrogen levels in men and women may be one possible explanation for the sex difference. Additionally, a reduction in estrogen levels after menopause in women may result in dysregulation of blood glucose and lipid metabolism [38], thereby increasing the risk of T2DM among women. The SLC2A9 gene may modulate the association between hyperuricemia and diabetes [37] and have a higher effect on SUA levels in women than in men [39], which possibly suggests a genetic basis for the sex differences. However, more studies are warranted to explore the mechanism of the sex-specific association between SUA level and T2DM risk.

This retrospective cohort analysis with a large sample size and well-measured covariates provides strong evidence for the sex-specific associations of the baseline SUA level and its dynamic change with T2DM. However, limitations should be considered when deriving conclusions. First, we could not estimate the effect of unmeasured confounders such as smoking, alcohol consumption, physical activity, and family history of diabetes on the association between SUA level and diabetes risk. Second, the change in hyperuricemia status was based on measurements from only baseline and one follow-up, and we failed to identify the reasons for the changes. Further studies should explore the effectiveness of medical treatment or lifestyle modification to decrease the elevation of SUA levels to prevent or reduce the risk of T2DM, especially among women. Third, we did not collect information on medications to lower SUA levels, so we cannot understand how the medication affects the association between the dynamic status of SUA level and T2DM risk. Fourth, the diagnosis was made at follow-up, so whether the change in hyperuricemia status occurred before or after T2DM development remains uncertain. Therefore, the causal relation between the transformation of hyperuricemia status and T2DM risk could not be established. Finally, most participants were employees of local governmental organizations in China, so the findings might be representative of highly educated and employed people only.

In this large cohort study, we found that higher levels of SUA were associated with an increased risk of T2DM only among women. Additionally, our findings provide epidemiological evidence to better understand the effect of the change in hyperuricemia status on the risk of T2DM. The findings stress the importance of monitoring SUA levels and maintaining them in a normal range for preventing or reducing incident T2DM in Chinese women.

## Figures and Tables

**Table 1:** Baseline characteristics of the study population according to baseline serum uric acid quartile by sex.

Baseline characteristics	SUA quartiles				*P*
	Q1 (*n* = 10,058)	Q2 (*n* = 10,095)	Q3 (*n* = 10,021)	Q4 (*n* = 10,022)	
Men					
SUA range (mg/dL)	≤5.42	5.42-6.20	6.20-7.05	>7.05	
*n*	5,282	5,333	5,296	5,295	
Age (years)	44.03 (12.83)	42.9 (12.62)	42.89 (12.91)	42.59 (12.67)	<0.001
Heart rate (beats/min)	75.02 (9.9)	75.21 (9.65)	75.43 (9.72)	76.35 (10.37)	<0.001
BMI (kg/m^2^)	24.78 (3.13)	25.5 (3.13)	26.22 (3.06)	27.12 (3.16)	<0.001
Height (cm)	171.45 (5.76)	172.06 (5.7)	172.27 (5.77)	172.51 (5.84)	<0.001
Weight (kg)	72.88 (10.19)	75.58 (10.46)	77.89 (10.37)	80.82 (10.99)	<0.001
WC (cm)	86.21 (8.42)	88.01 (8.53)	89.82 (8.11)	92.21 (8.35)	<0.001
Blood pressure (mmHg)					
Systolic	121.24 (15.21)	121.54 (14.9)	123.07 (14.57)	124.54 (14.63)	<0.001
Diastolic	75.64 (10.25)	76.59 (9.74)	77.53 (9.95)	79.19 (9.95)	<0.001
SUA (mg/dL)	4.81 (0.52)	5.84 (0.22)	6.63 (0.24)	7.98 (0.79)	<0.001
FPG (mmol/L)	5.27 (0.52)	5.28 (0.51)	5.33 (0.51)	5.37 (0.53)	<0.001
TC (mmol/L)	4.72 (0.87)	4.81 (0.89)	4.92 (0.91)	5.08 (0.95)	<0.001
TG (mmol/L)	1.47 (1.15)	1.67 (1.25)	1.88 (1.37)	2.32 (1.79)	<0.001
HDL-C (mmol/L)	1.32 (0.29)	1.27 (0.27)	1.24 (0.26)	1.21 (0.26)	<0.001
LDL-C (mmol/L)	2.92 (0.72)	2.99 (0.74)	3.08 (0.73)	3.15 (0.77)	<0.001
eGFR (mL/min/1.73 m^2^)	97.49 (19.05)	93.96 (17.45)	91.91 (16.81)	88.94 (16.53)	<0.001
White blood cell					
ALT (IU/L)	20.00 (15.00-27.00)	22.00 (16.80-30.10)	23.40 (17.70-34.00)	28.00 (19.93-40.10)	<0.001
Women					
SUA range (mg/dL)	≤3.77	3.77-4.37	4.37-5.05	>5.05	
*n*	4,080	3,966	4,030	4,014	
Age (years)	37.79 (9.78)	38.3 (10.71)	39.67 (12.05)	43.58 (14.04)	
Heart rate (beats/min)	76.8 (9.9)	76.63 (10)	76.28 (9.75)	76.02 (9.82)	<0.001
BMI (kg/m^2^)	22.28 (2.87)	22.78 (3.09)	23.46 (3.34)	24.98 (3.72)	<0.001
Height (cm)	160.48 (5.23)	160.82 (5.33)	160.72 (5.37)	160.22 (5.46)	<0.001
Weight (kg)	57.36 (7.74)	58.9 (8.21)	60.55 (8.8)	64.08 (9.94)	<0.001
WC (cm)	72.68 (7.22)	74.17 (7.7)	75.85 (8.35)	79.75 (9.19)	<0.001
Blood pressure (mmHg)					
Systolic	110.21 (14.02)	110.77 (14.74)	112.08 (15.06)	116.89 (16.1)	<0.001
Diastolic	68.43 (9.05)	69.41 (9.53)	70.25 (9.65)	73.16 (9.92)	<0.001
SUA (mg/L)	3.35 (0.35)	4.09 (0.17)	4.69 (0.19)	5.78 (0.67)	<0.001
FPG (mmol/L)	5.01 (0.42)	5.04 (0.44)	5.08 (0.46)	5.2 (0.52)	<0.001
TC (mmol/L)	4.5 (0.85)	4.6 (0.87)	4.75 (0.9)	5.02 (0.97)	<0.001
TG (mmol/L)	0.94 (0.55)	1.02 (0.65)	1.13 (0.72)	1.43 (0.92)	<0.001
HDL-C (mmol/L)	1.58 (0.34)	1.55 (0.34)	1.51 (0.33)	1.44 (0.32)	<0.001
LDL-C (mmol/L)	2.6 (0.68)	2.7 (0.7)	2.82 (0.74)	3.07 (0.79)	<0.001
eGFR (mL/min/1.73 m^2^)	113.28 (24.91)	107.47 (23.54)	103.33 (23.11)	97.5 (22.36)	<0.001
White blood cell					
ALT (IU/L)	13.00 (10.08-16.90)	13.20 (11.00-17.70)	14.00 (11.00-19.00)	17.00 (12.70-23.60)	<0.001

Abbreviations: BMI: body mass index; WC: waist circumference; SUA: serum uric acid; FPG: fasting plasma glucose; TC: total cholesterol; TG: triglycerides; HDL-C: high-density lipoprotein cholesterol; eGFR: estimated glomerular filtration rate; ALT: alanine aminotransferase. Data are mean (SD) and median (interquartile range). Quartiles 1-4: ≤5.44, >5.44~≤6.22, >6.22~≤7.08, and >7.08 mg/dL for men and ≤3.80, >3.77~≤4.38, >4.38~≤5.05, and >5.05 mg/dL for women.

**Table 2:** Association between baseline serum uric acid and risk of type 2 diabetes mellitus.

	Person-year	Incident diabetes	Incidence (per 1,000 person-years)	Model 1^†^	Model 2^‡^	Model 3^§^	
Men							
Q1	19,509	300	15.38	1.00	1.00	1.00	
Q2	20,080	334	16.63	1.13 (0.97-1.32)	1.05 (0.87-1.27)	1.09 (0.90-1.31)	
Q3	19,738	362	18.34	1.21 (1.04-1.41)	1.01 (0.84-1.22)	1.04 (0.87-1.26)	
Q4	19,449	441	22.67	1.54 (1.33-1.78)	1.12 (0.93-1.35)	1.11 (0.92-1.34)	
*P*_trend_				<0.001	0.249	0.371	
Hyperuricemia	20,941	479	22.87	1.41 (1.27-1.58)	1.12 (0.98-1.29)	1.06 (0.92-1.22)	
SUA (per 1-mg/dL)	78,776	1,678	21.30	1.14 (1.10-1.19)	1.04 (0.98-1.09)	1.04 (0.99-1.10)	
Women							
Q1	13,193	48	3.64	1.00	1.00	1.00	
Q2	13,151	62	4.71	1.21 (0.83-1.76)	1.03 (0.65-1.65)	1.00 (0.62-1.61)	
Q3	13,424	125	9.31	2.10 (1.50-2.94)	1.70 (1.12-2.58)	1.78 (1.17-2.71)	
Q4	13,868	270	19.47	3.37 (2.46-4.62)	2.14 (1.43-3.22)	1.93 (1.27-2.93)	
*P*_trend_				<0.001	<0.001	<0.001	
Hyperuricemia	3,906	120	30.72	2.40 (1.94-2.97)	1.82 (1.38-2.40)	1.35 (1.01-1.80)	
SUA (per 1-mg/dL)	53,637	585	10.91	1.55 (1.44-1.68)	1.31 (1.17-1.45)	1.20 (1.08-1.34)	

Abbreviations: BMI: body mass index; WC: waist circumference; SUA: serum uric acid; FPG: fasting plasma glucose; TC: total cholesterol; TG: triglycerides; HDL-C: high-density lipoprotein cholesterol; eGFR: estimated glomerular filtration rate; ALT: alanine aminotransferase. Data are hazard ratios (HRs) (95% confidence intervals (CIs)). ^†^Model 1: adjusted for age at baseline. ^‡^Model 2: adjusted for age, BMI, WC, heart rate, systolic blood pressure, and levels of TC, TG, HDL-C, eGFR, ALT, and white blood cell count at baseline. ^§^Model 3: adjusted for FPG level at baseline, plus all variables in model 2.

**Table 3:** Association between transformation of serum uric acid levels and risk of type 2 diabetes mellitus.

Hyperuricemia at baseline	Hyperuricemia at follow-up	No. of participants	Cases	Mean follow-up duration (months)	Model 1^†^	Model 2^‡^	Model 3^§^
Men							
No	No	13,210	838	44.12	1.00	1.00	1.00
Yes	No	1,913	187	44.54	1.62 (1.36-1.92)	1.1 (0.88-1.36)	1.07 (0.83-1.36)
No	Yes	2,296	120	48.43	0.97 (0.79-1.19)	0.75 (0.58-0.95)	0.85 (0.65-1.1)
Yes	Yes	3,787	292	43.86	1.37 (1.19-1.58)	0.82 (0.68-0.98)	0.88 (0.72-1.08)
Women							
No	No	14,106	322	39.56	1.00	1.00	1.00
Yes	No	571	38	41.28	2.08 (1.43-2.96)	0.96 (0.56-1.57)	0.77 (0.43-1.32)
No	Yes	845	63	45.88	2.69 (2.00-3.58)	1.59 (1.07-2.31)	1.67 (1.09-2.49)
Yes	Yes	568	82	41.02	3.94 (2.97-5.19)	2.25 (1.56-3.19)	2.15 (1.44-3.16)

Abbreviations: BMI: body mass index; WC: waist circumference; SUA: serum uric acid; FPG: fasting plasma glucose; TC: total cholesterol; TG: triglycerides; HDL-C: high-density lipoprotein cholesterol; ALT: alanine aminotransferase. Data are odds ratios (ORs) (95% confidence intervals (CIs)). ^†^Model 1: adjusted for age at baseline and follow-up. ^‡^Model 2: adjusted for age, BMI, WC, heart rate, systolic blood pressure, and levels of TC, TG, HDL-C, eGFR, ALT, and white blood cell count at baseline and follow-up. ^§^Model 3: adjusted for FPG level at baseline plus all variables in model 2.

## Data Availability

The datasets used and/or analyzed during the current study are available from the corresponding author on reasonable request.
